# Logistics of Implementing a Large-scale Typhoid Vaccine Trial in Kathmandu, Nepal

**DOI:** 10.1093/cid/ciy1125

**Published:** 2019-03-07

**Authors:** Rachel Colin-Jones, Mila Shakya, Merryn Voysey, Katherine Theiss-Nyland, Nicola Smith, Dikshya Pant, Xinxue Liu, Susan Tonks, Olga Mazur, Yama G Farooq, Sarah Kelly, Anup Adhikari, Sabina Dongol, Abhilasha Karkey, Shrijana Shrestha, Buddha Basnyat, Andrew J Pollard

**Affiliations:** 1 Oxford Vaccine Group, Department of Paediatrics, University of Oxford, United Kingdom; 2 Oxford University Clinical research Unit – Nepal; 3 Paediatric Research Unit, Patan Hospital; 4 Nepal Family Development Foundation, Kathmandu

**Keywords:** typhoid vaccine, randomized control trial, implementation, logistics, Nepal

## Abstract

Typhoid fever is estimated to affect over 20 million people per year worldwide, with infants, children, and adolescents in south-central and southeast Asia experiencing the greatest burden of disease. The Typhoid Vaccine Acceleration Consortium (TyVAC) aims to support the introduction of typhoid conjugate vaccines into Gavi-eligible countries in an effort to reduce morbidity and mortality from typhoid. TyVAC-Nepal is a large-scale, participant- and observer-blind, individually randomized, controlled trial evaluating the efficacy of a newly developed typhoid conjugate vaccine in an urban setting in Nepal. In order to effectively deliver the trial, a number of key elements required meticulous planning. Public engagement strategies were considered early, and involved the implementation of a tiered approach. Approximately 300 staff were employed and trained in order to achieve the mass vaccination of 20 000 children aged 9 months to ≤16 years old over a 4-month period. There were 19 vaccination clinics established across the Lalitpur metropolitan city in the Kathmandu valley. Participants will be followed for 2 years post-vaccination to measure the rate reduction of blood culture–confirmed typhoid fever in the vaccination arm as compared to the control arm. The experience of conducting this large-scale vaccine trial suggests that comprehensive planning, continuous monitoring, and an ability to adapt plans in response to feedback are key.

In order to reduce the morbidity and mortality caused by *Salmonella enterica* serovar Typhi (*S.* Typhi), the Typhoid Vaccine Acceleration Consortium (TyVAC) aims to support the introduction of a typhoid conjugate vaccine into Gavi-eligible countries [1]. To generate evidence on the impact of a newly developed typhoid conjugate vaccine (Vi-TCV), 3 vaccination trials are being conducted in Nepal, Bangladesh, and Malawi; the trial discussed in this article is TyVAC-Nepal.

TyVAC-Nepal is a participant- and observer-blind, individually randomized, controlled trial to evaluate the efficacy of Vi-TCV in Nepali children aged 9 months to <16 years of age [2]. All children will be followed for 2 years post-vaccination to determine the efficacy of Vi-TCV in an endemic population setting, measured as the rate reduction of blood culture–confirmed typhoid fever in the vaccination arm as compared to the control arm [2].

We aimed to enroll 20 000 children over a 4-month period. Children enrolled were randomized in a 1:1 ratio to receive either Vi-TCV or the control vaccine, a Group A meningococcal vaccine.

Prior to vaccination and dependant on consent, 1500 participants were also randomized to a sub-study investigating the immunogenicity of Vi-TCV and the persistence of antibodies. Baseline blood samples were collected at the first study visit, then at 28 days, 18 months, and 24 months post vaccination [2].

Following ethical and regulatory approval, a comprehensive plan was developed for the delivery and management of the trial. This article presents the logistics of implementing this plan.

## VACCINATION CAMPAIGN/DELIVERY

### Public Engagement

**Figure 1.**
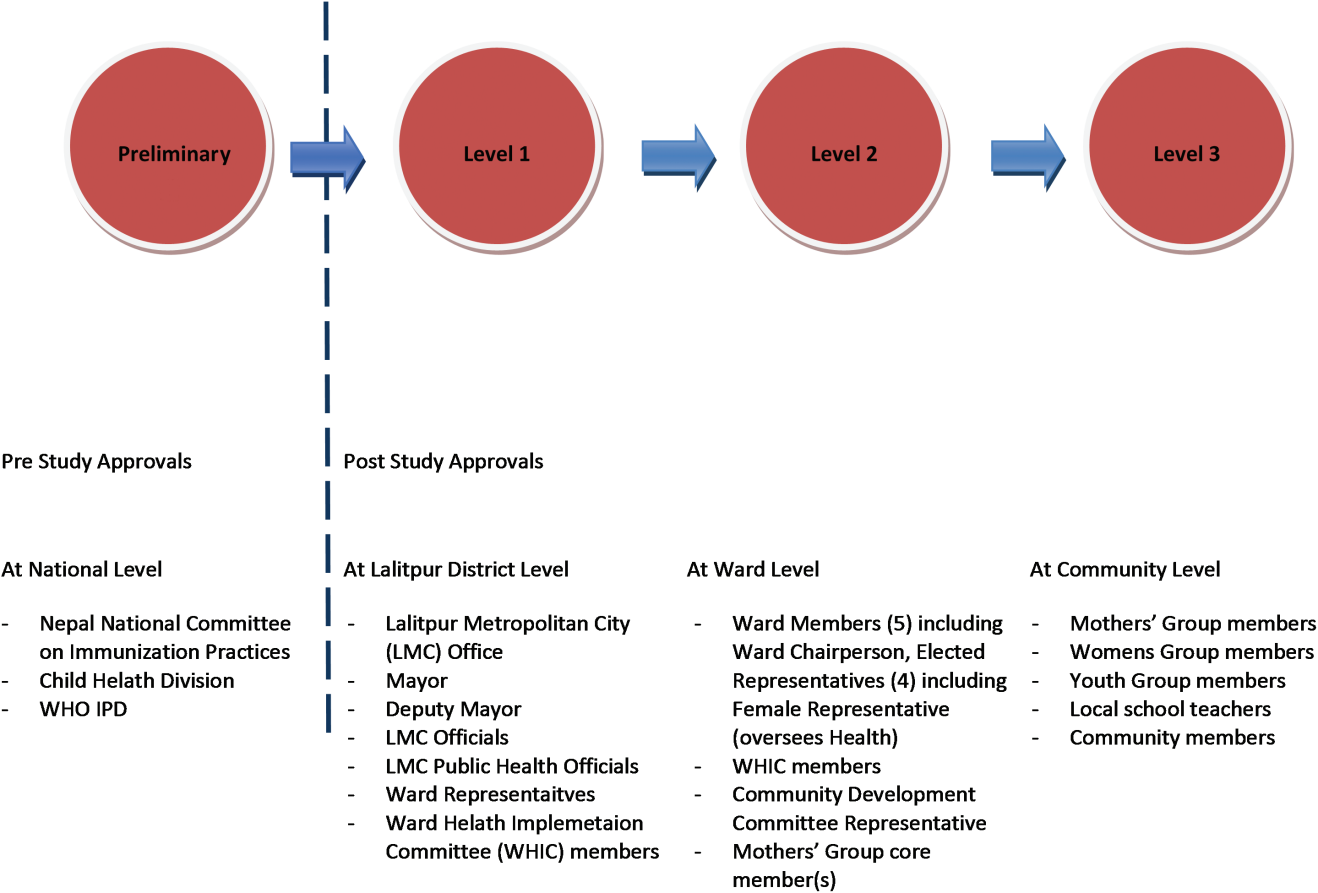
Tiered approach to public engagement in TyVAC-Nepal. Stages required, and groups approached during public engagement in TyVAC-Nepal. Abbreviations: LMC, Lalitpur Metropolitan City; TyVAC, Typhoid Vaccine Acceleration Consortium; WHIC, Ward Health Implementation Committee; WHO IPD, World Health Organization – Immunization Preventable Diseases.

The importance of public engagement to the success of any clinical trial involving human participants is well documented [3–6]. Figure 1 depicts the tiered approach to public engagement that was taken in TyVAC-Nepal. Prior to the trial starting, the National Committee on Immunisation Practices (the Nepalese national immunization technical advisory group), along with the Child Health Division Ministry of Health, Nepal, and other national-level stakeholders, were engaged and informed about the planned activities. As a first step to engaging the community, immediately following ethical and regulatory approval, the project team held meetings with the local government and municipal committees at a district level and obtained approvals to approach community stakeholders. Independent meetings were then held with the local governments of individual wards (municipality-defined geographical areas), and a tiered approach was taken to engage the public in each of the wards. At the first level, publicly elected ward committee members, the ward health implementation committee, and other key decision-makers were approached to obtain permission to engage community members and to set up the vaccination clinics. Following agreement, Community Development Committees, Tole (community) Heath Promoters (THPs), community leaders, and teachers were invited to engage in discussions about planned trial activities. Lastly, the project team visited community members, mothers’ groups, and other local groups to disseminate study information.

Throughout the vaccination period, THPs selected by the ward authorities conducted door-to-door visits and distributed participant information leaflets. THPs are experienced, local, health-care volunteers, who know the families in their catchment area and play important roles in various health-related programs, including national vaccine campaigns.

Public engagement is an ongoing process, and updates on trial activities continue to be given regularly. Ongoing engagement is sustained via meetings with local stakeholders and community members, conducted with the purpose of disseminating information and maintaining a continuous dialogue with the community. In addition, the public engagement sessions provide an opportunity for the project team to understand community perspectives and opinions.

### Staff Training

In order to achieve a mass vaccination campaign that enrolled and vaccinated 20 000 children in 4 months, alongside other trial activities, approximately 300 staff were hired and trained (Table 1).

**Table 1.** Staff Group Breakdown

Role	Number of Staff Required
Project manager	1
Pediatrician	1
Field team manager	1
Junior doctors	7
Clinic supervisors	15
Counselors	22
Clinically qualified staff (nurses/health assistants/vaccinators)	100
Communication assistants	10
Data and IT assistants	5
Vaccine logistics officers	2
Quality assurance officer	1
Public engagement officer	1
Sample and equipment transport assistants	2
THPs (local community health workers)	154

Abbreviations: IT, information technology; THP, Tole (community) Heath Promoters.

Only 7 members of staff had previously worked in research, necessitating a training program that covered a wide range of topics and started from basics. This provided the scope for capacity building and individual professional development, an opportunity embraced by both trainers and trainees.

**Figure 2.**
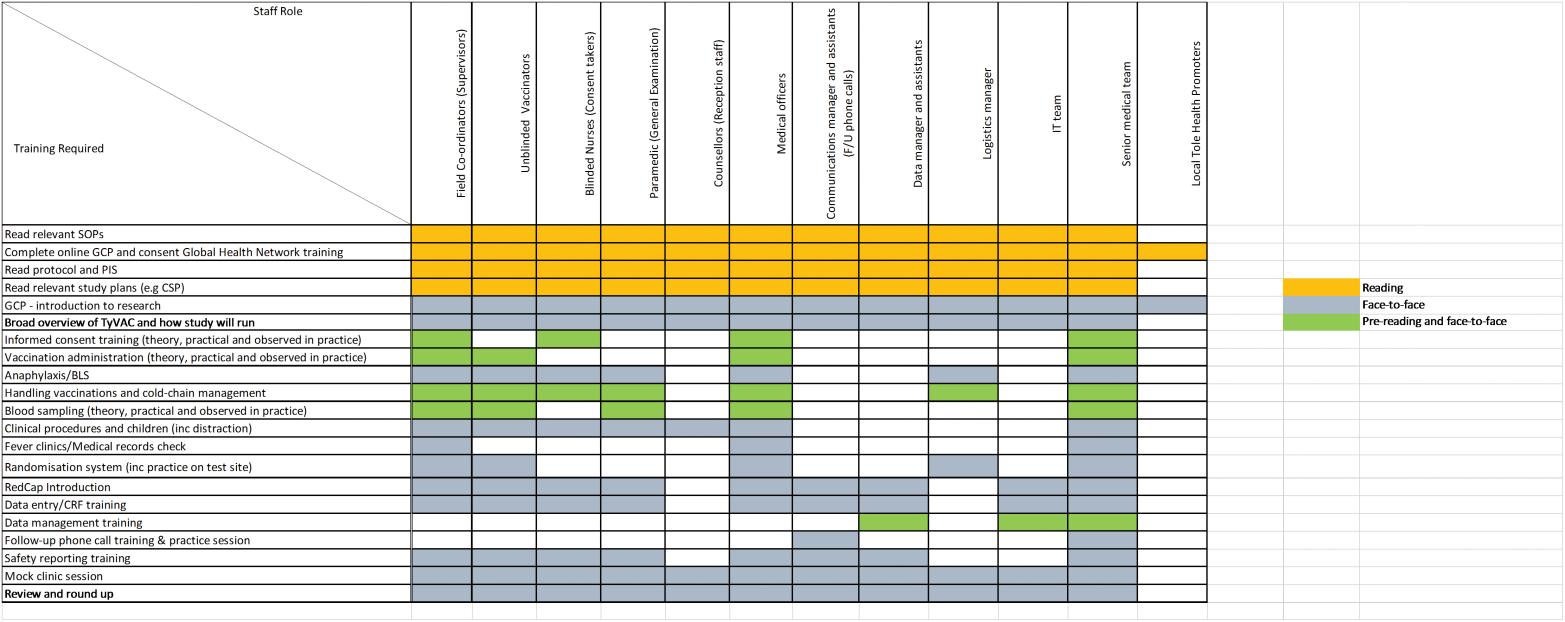
Staff training matrix. Training required according to staff role, prior to the delivery of TyVAC-Nepal. Abbreviations: BLS, basic life support; CRF, case report form; CSP, Clinical Study Plan; F/U, follow-up; GCP, good clinical practice; IT, information technology; PIS, participant information sheet; SOP, standard operating procedure; TyVAC, Typhoid Vaccine Acceleration Consortium.

Figure 2 is a training matrix showing the modules covered for each staff group. The majority of training sessions were interactive and facilitated in small groups, in order to promote an environment where questions could be asked. Following theoretical and practical sessions, clinical staff, whose role would include obtaining consent, venepuncture, and administering vaccinations, spent time in an established clinic observing more-experienced practitioners. Prior to staff members working autonomously, they were observed in practice and had to pass a competency assessment. In order to achieve this, 12 assessors were trained to evaluate competence in the field and vaccination clinic openings were staggered to ensure each team was able to spend time in an already-functioning clinic prior to running their own.

Following the training program, vaccination campaign staff were divided into 15 teams, each made up of 1 supervisor, 1 counselor, 1 health assistant, 1–2 consenters, and 3–4 unblinded vaccinators. A team of 7 medical officers had oversight of 2–3 clinics each. Oversight included assessments of clinical staff competence, provision of medical input during eligibility screening, and working closely with the clinic supervisors to ensure the clinics operated according to the protocol and clinical study plan. Additional staff contributing to the delivery of the vaccination campaign included the project management, information technology/data management, vaccine logistics, quality assurance, transport, and laboratory teams.

### Vaccination Clinics

**Figure 3.**
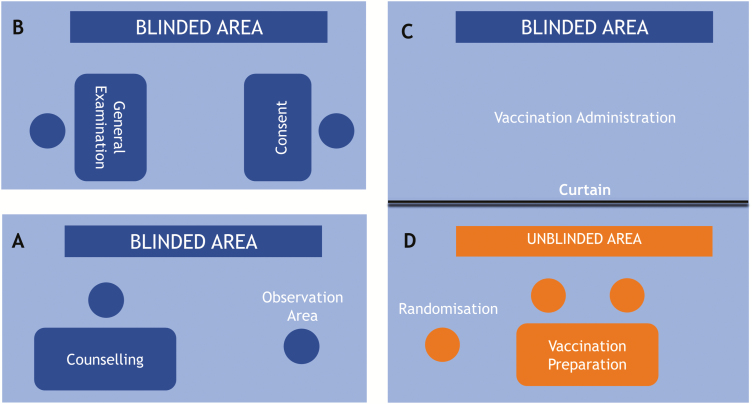
Clinic set-up and maintaining blinding. Demonstration of how clinics were set up; blinding was maintained using a curtain during the vaccination campaign.

The project management team worked with local community stakeholders and each clinic supervisor to identify space in which a clinic could be established. In order to prioritize participant and staff safety, maintain blinding, and deliver trial activities efficiently, key minimum requirements were identified. It was important that the clinic was easily and safely accessible; had working electricity; provided spaces where vaccination and venepuncture could happen, with adequate room for appropriate clinical holding of children and handling of sharps; and that the space was big enough to divide into 4 areas (Figure 3), 1 of which would be an unblinded area hidden behind a curtain (Figure 3D). Over 50 spaces were viewed, with 20 identified as both suitable and available at the required time. The spaces used included community clinic buildings, schools, basement garages, and, for 1 clinic, a basic corrugated iron structure. Generally, 1–2 clinics were established per ward, and consideration was given to their location in relation to the geographical boundaries of that ward.

None of the clinic spaces had Internet access. Due to the high volume of data being generated, an electronic system was essential in order to mitigate against the logistical challenges of transporting, managing, and storing large numbers of paper source documents. Electronic, direct data entry reduces the chance of errors being made, as system logic and real-time validations can be built into the data collection tools, thus promoting higher-quality data capture [7]. To achieve this, portable servers accessible through an Intranet were developed in each clinic, and data were collected on laptops connected to this local network. At the end of each day, the local server was transported to a facility with a secure Internet connection and the data collected during that day were uploaded onto the main server. This allowed for the daily monitoring of data quality and for timely data cleaning.

THPs visited households and invited parents/guardians to vaccination clinics. On arrival, participants were welcomed by a counselor, who would ensure that parents/guardians and participants had read the participant information booklet, which included information on typhoid, the study plan, and when to attend the typhoid surveillance clinics post-vaccination (Figure 3A). If participants and parents/guardians had not read the participant information booklet yet, they were encouraged to do so at this point or, if illiterate, the information was read to them. Time was given to answer any questions prior to the participant moving on to see the nurse, who would obtain parental consent. In children aged >7 years, assent was also obtained (Figure 3B).

If the participant’s parent/guardian was illiterate, an independent witness was present throughout the consent process to verify an adequate explanation of the trial. In order to ensure consistency and completeness, all staff obtaining consent followed a pre-defined consent script. This included the key messages from the information sheet in simple, culturally appropriate language that had been cross-checked and verified by multiple native speakers prior to use in the trial. Soon after starting, it was noted that parents/guardians had many questions about the reason for blinding, whether this was an “experiment” on Nepali children, and who would be held responsible if a child died due to the vaccine. To address these questions systematically, a frequently asked question and answer document was developed and used at the point of consent and, more widely, across trial activities to equip staff to deal with difficult questions. The concept of a participant- and observer-blind, individually randomized, controlled trial and the reasons that randomization and maintaining blinding are important were covered with each parent/guardian during the consent process. Further, participants and parents/guardians had the opportunity to ask questions and clarify their understanding. As well as answering questions during the consent process, ongoing dialogues and liaisons with local stakeholders and community leaders helped maintain public confidence and address questions and concerns more broadly.

Following consent, participants were screened (Figure 3B) and had an abbreviated general examination to ensure eligibility. Provided there were no eligibility concerns, participants were then seen by the unblinded team (Figure 3C). A purpose-built, offline mobile application was used to randomize participants for both the main study and the immunogenicity study. To maintain blinding, randomization and vaccine preparation took place behind a curtain (Figure 3D), where only unblinded staff were permitted. Moving forward, unblinded staff have not been and will not be involved in any follow-up contacts for participants that they vaccinated.

If a participant consented and was randomized to the immunogenicity sub-study, a blood sample would be obtained prior to vaccination. Local anaesthetic creams, which are used widely in many countries to reduce procedural pain [8], are not licenced in Nepal. However, distraction is a simple and effective non-pharmacological pain management technique that significantly reduces procedural pain and distress [9]. The aim of distraction is to shift the child’s focus away from the painful stimuli to something engaging and absorbing, thus reducing pain, anxiety, and distress [9]. All unblinded staff were trained in clinical procedures with children, including distraction techniques. The specific techniques used depended on the age of the child, but included multisensory toys, interactive books, breathing games, and dynamic technology.

Following vaccination, participants were observed for immediate adverse events for a minimum of 15 minutes. All clinical staff were trained in anaphylaxis and basic life support, and both first- and second-line emergency kits were available in each clinic. None of the clinical staff had previously received formal anaphylaxis training. Because this is a skill that is rarely used, but needs to be maintained, regular refresher training was given during the vaccination campaign. No immediate serious adverse events were observed.

**Figure 4.**
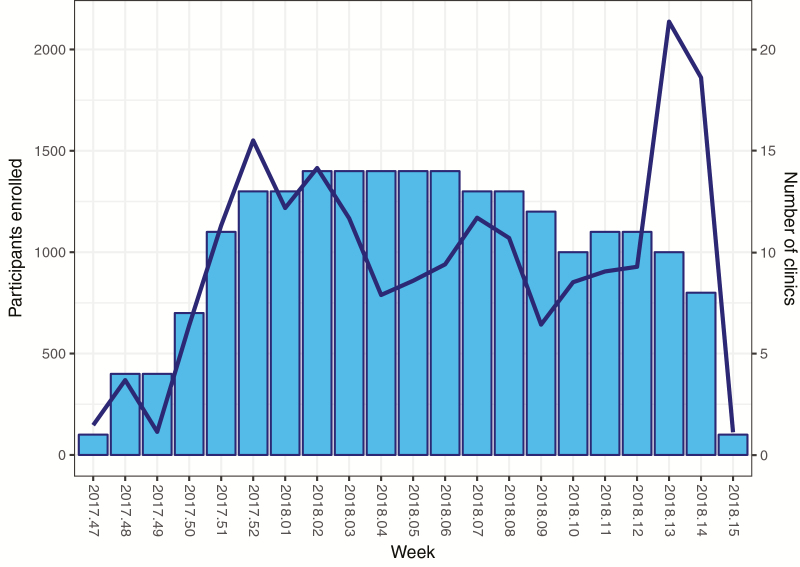
Weekly recruitment over time. Participants enrolled per week and the corresponding number of vaccination clinics open at the time.

The original plan was to operate 15 clinics for the duration of the 4-month recruitment phase. However, clinics recruited at different rates and recruitment dropped over time as the proportion of children vaccinated in each ward increased. Real-time monitoring of recruitment rates, age distributions, and blood draw successes per clinic was conducted weekly. This information contributed to the development of bespoke recruitment and training plans that were updated and adapted regularly throughout the vaccination campaign, in close communication with each clinic team. For example, in January 2018, despite having all the planned clinics established, the weekly recruitment plots showed that enrollment was starting to taper. The project management team worked with each clinic to identify reasons for the slowing recruitment in their ward and, in response, implemented bespoke plans that boosted recruitment rates. After a further month, enrollment slowed for a second time, prompting new strategies to be adapted, again increasing recruitment (Figure 4).

Recruitment strategies included establishing additional clinics in wards that were geographically large and early closures of clinics in smaller wards, to more efficiently employ teams elsewhere. In some larger wards, it was difficult to identify new, permanent clinic spaces and, therefore, teams operated mobile clinics on Saturdays, focusing on areas that either were geographically far from the permanent clinic or had particularly low recruitment rates.

In week 13, clinics were opened in 2 large wards where the communities were eager to participate in the trial, thus resulting in the recruitment peak seen in Figure 4.

On 9 April 2018, target enrollment was completed, with 20 019 children vaccinated in just over 4 months.

## PASSIVE SURVEILLANCE

The primary outcome measure of this trial relies on ascertaining the incidence of blood culture–confirmed typhoid fever in both of the vaccination arms. Successfully obtaining blood cultures is, therefore, an essential element of the trial. Some basic principles apply to the process, such as the use of an aseptic technique and the careful use of antiseptic substances [10–12]. The use of blood culture for detection of *S.* Typhi is notoriously problematic, with sensitivity estimates ranging between 40–80% [13, 14]. A contributing factor is the volume of blood cultured: a Vietnamese study demonstrated that half of patients with blood culture–positive typhoid fever had less than 1 colony-forming unit/millilitre, meaning that larger volumes of blood are more likely to yield results [15]. For this reason, it was stipulated in TyVAC-Nepal that 4 ml of blood should be taken for culture when possible: a practice which has been regularly audited and reviewed.

The passive surveillance element of TyVAC-Nepal is designed to capture as many blood cultures from potential typhoid fever cases as possible. For this purpose, 16 community-based passive surveillance clinics and 1 hospital-based clinic have been established. Medically qualified doctors run all passive surveillance clinics, and the facilities are equipped to treat/triage all patients who present. Treatment decisions are made based on an initial clinical assessment and blood investigations (including cultures), once available.

Parent/guardians of participants are encouraged to bring their children to these clinics if the child either has a current temperature of ≥38°C and/or a self-reported fever of ≥2 days. These criteria were selected as a result of a previous study from Nepal, which showed that *S.* Typhi could be cultured from blood early in a febrile illness, in many cases without the accompanying symptoms typical of enteric fever [16]. The utilization of such stringent criteria should improve the detection of true, positive cases.

If 1 of the fever criterion is met, consent is obtained to take a blood sample for culture. Routine investigations, including the blood culture and treatment, are free for TyVAC participants. No further financial incentives are given, and would not be culturally acceptable.

**Figure 5.**
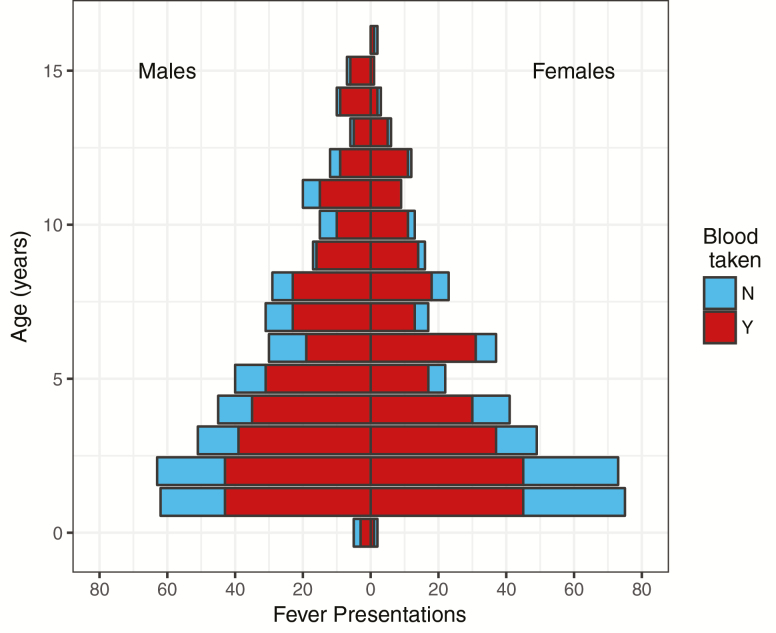
Eligible trial participants presenting to fever clinics. Age distribution of eligible* trial participants presenting to fever clinics during the first 6 months of follow-up. *Vaccinated in the TyVAC-Nepal trial and with current temperature ≥38°C or self-reported at least 2 days of fever.

Participants under 5 years of age represent the highest proportion of eligible children presenting to passive surveillance clinics (Figure 5). High rates of blood draw refusal (Figure 5) present an ongoing challenge. Reported reasons for refusal include wanting to try medication first; parents feeling the duration of the fever is too short or the child is too young to warrant a blood sample; and a fear of children becoming weak. This is currently being addressed through public engagement and education; the use of distraction techniques; and the introduction of incentives for children, such as coloring books and pencil cases.

## DISCUSSION

To deliver a large clinical trial, like TyVAC-Nepal, the clinical trial team has to be proactive in their approach to implementation, whilst being dynamic in responding to a changing environment. Specifically, 3 areas in which specific adaptations are needed include public engagement, training, and recruitment management.

### Public Engagement

There is a fundamental need for public engagement in order to successfully conduct clinical research involving human participants, including sound and robust communication with the community [3, 5, 6, 17, 18]. In 3 Phase I human immunodeficiency virus trials in India, Sahay et al [4] identified that having a multi-level participatory approach to public engagement was essential in establishing and maintaining community participation. TyVAC-Nepal, similarly, used a tiered approach to public engagement, with success at each level being dependent on understanding and collaboration at the previous level. It was found to be important, at an early stage, to engage political leaders, key stakeholders, community decision-makers, and members of the community in an ongoing dialogue about trial activities.

Public engagement in TyVAC-Nepal has been a continuous and dynamic process. Being receptive to thoughts and concerns from within the community enabled the responsive adaptation of activities, messages, and resources [19, 20]. Similarly, an Ebola vaccine trial conducted in Liberia found that a community mobilization strategy that proactively reviewed the output and impact of public engagement enabled the early identification and adaption of the strategy in order to address any existing gaps [21]. A willingness to adapt activities and messages, as opposed to sticking rigidly to preconceived plans and notions, has been instrumental in building trust and positive relationships in TyVAC-Nepal.

Working with THPs helped to develop and maintain community confidence. Involving the local THPs allowed community leaders to have a level of participation in the study and helped to establish trust more generally within these communities. They were key to public engagement efforts, as they had established networks in their communities. Sahay et al [4] also worked with community health volunteers in India, and recognized this as pivotal to establishing networks and building trust in their catchment areas. In a rotavirus vaccine trial in Bangladesh, working with community health workers was key to engaging members of the public and building long-standing, positive relationships with the community, thus allowing further research to be conducted in the area [5].

### Training

Training prior to the start of TyVAC-Nepal was necessary; however, ongoing education throughout the implementation phase of the trial was required to maintain standards, refresh newly learned skills, and train new starters (employed due to staff attrition). Several other studies have also highlighted the need for staff training to be an ongoing, adaptive, and responsive process, as gaps in trial staff knowledge or experience are identified [22–25].

In delivering an Ebola vaccine trial in Sierra Leone, Carter et al [25] proactively implemented weekly meetings where staff were trained or had refresher training on topics that were self-identified as knowledge deficits. Although retraining needs were identified in real time in TyVAC-Nepal, via supervisors and monitoring structures, future projects would benefit from Carter et al’s [25] proactive approach to the identification of learning needs in the staff groups. An awareness of the need for and preplanning for ongoing training would enable enhanced preparedness and a rapid response to needs.

Investment in a robust training structure when delivering clinical trials has been found to support the delivery of high-quality trials and to provide the opportunity for both individual and health-service capacity building [6, 22, 24]. Ongoing learning and development are beneficial for both clinical trials and individuals, and creating a team environment where these are valued can enable retraining to be seen positively as standard practice [20, 22–24].

### Recruitment Management

Although a recruitment strategy was preplanned in TyVAC-Nepal, changes were continuously required to ensure the timely enrollment of the participants. Electronic data entry, with daily uploads, combined with data outputs in graphical formats, resulted in the central study team being able to continuously monitor recruitment by each clinic in real time. This enabled the team to detect when recruitment was slowing, develop and institute remedial measures, and then monitor for effectiveness. Geldenhuys et al [23] also found considerable value in utilizing data for monitoring enrollment rates in a Phase IV poliomyelitis vaccine trial conducted in South Africa.

Using graphical data outputs could identify such things as slowing recruitment; however, this needed to be coupled with continuous community engagement by ground staff, to ascertain the reasons for varying recruitment rates [21, 26]. Understanding root causes meant the team could adapt to local circumstances, utilize the dedicated public engagement team as needed, and update the central team so that wider plans could be altered as necessary. Enria et al [27] highlight that having an understanding of the community dynamics that were feeding into recruitment strategies was invaluable in an Ebola vaccine trial in Sierra Leone.

Adaptability, with respect to geographical area, physical clinic spaces, opening times, and team mobility, was vital in maintaining recruitment rates. Open-minded attitudes, lateral thinking, and flexibility of the wider team made it possible to drastically alter preconceived ideas about how the recruitment phase would unfold.

## CONCLUSION

In conducting this large-scale vaccine trial, experience has suggested that comprehensive planning, continuous monitoring, and an ability to adapt plans in response to feedback have been key. Taken with expert opinions, the logistical elements of implementation discussed in this manuscript may be important for other researchers in the planning and delivery of similar large-scale trials in the future.

## Notes

***Author contributions.*** R. C.-J. and M. S. led the trial implementation the development of this paper. A. A. managed the field teams during trial implementation. M. V. analyzed the data. K. T.-N. provided scientific oversight. N. S. and D. P. provided clinical management for the trial. B. B., S. S., and A. J. P. supervised the work. All authors reviewed and approved the final version of the paper.

***Financial support.*** This publication is based on research funded in part by a grant from the Bill & Melinda Gates Foundation (OPP1151153).

***Supplement sponsorship.*** This supplement is sponsored by the Center for Vaccine Development and Global Health (CVD) at the University of Maryland School of Medicine.

***Potential conflicts of interest.*** A. J. P. previously conducted studies on behalf of Oxford University that were funded by vaccine manufacturers, but currently does not undertake industry-funded clinical trials; chairs the UK Department of Health and Social Care’s Joint Committee on Vaccination and Immunisation and the EMA scientific advisory group on vaccines; is a member of the World Health Organization’s Strategic Advisory Group of Experts; has received grants from the Wellcome Trust, Bill & Melinda Gates Foundation, Medical Research Council, and NIHR Oxford Biomedical Research Centre, during the conduct of the study; and has received grants from Okairos and Pfizer, outside the submitted work. M. V. is funded by a NIHR Doctoral Research Fellowship (DRF-2015-08-048). All other authors report no potential conflicts. All authors have submitted the ICMJE Form for Disclosure of Potential Conflicts of Interest. Conflicts that the editors consider relevant to the content of the manuscript have been disclosed.
